# Supplementary material for: Content-rich biological network constructed by mining PubMed abstracts
Source: BMC Bioinformatics. 2004 Oct 8;5:147. doi: 10.1186/1471-2105-5-147 (PMC528731; doi:10.1186/1471-2105-5-147)
Supplement: Additional File 2 — The original results of the above study (non-essential files are deleted to keep the file size under the limit set by BMC bioinformatics). [file 1471-2105-5-147-S2.bz2 › chilibotAdditionalFile2/dip50/94ID9178909E401/html/CBL_CRK.html]

 


 **CBL** and **CRK** 
  
Found 57 abstracts in PubMed, retrieved 50.  
 

 What does Google say? 
 PDF only 
| .edu only 

---

**Interactive relationship** (e.g. stimulation, inhibition, etc)

**Stimulatory relationship**- In contrast to its constitutive interaction with Grb2, tyrosine phosphorylated  **Cbl**  only associates with  **Crk**  after BCR stimulation.  Ref: 8710377 Oncogene, 1996
- **Cbl**  b constitutively binds Grb2 and becomes associated with  **Crk**  L upon TCR stimulation.  Ref: 10022120 Oncogene, 1999
- Like c  **Cbl** ,  **Cbl**  b associates constitutively with CAP and interacts with  **Crk**  upon insulin stimulation.  Ref: 12842890 J Biol Chem< MedlineTA>J Biol Chem, 1999
- BCR ligation also increased the amount of  **Crk**  proteins in the particulate fraction of the cells and induced the formation of  **Crk** .Cas and  **Crk** . **Cbl**  complexes in the particulate fraction.  Ref: 8943292 J Biol Chem, 1996
- c  **Cbl**  and Nck are also tyrosine phosphorylated by cellular stimulation with hGH and associate with the p130 Cas CrkII  [ **CRK** ]  complex.  Ref: 9837978 J Biol Chem, 1998
- We also show that both  **Cbl**  and Gab1 enhance Met induced activation of another MAP kinase cascade, the ERK pathway, in a  **Crk**  independent manner.  Ref: 10962563 Oncogene, 2000
- The molecular adapter c  **Cbl**  is rapidly tyrosine phosphorylated following stimulation through the TCR and associates with Src homology domain 2 SH2 SH3 domain containing adapters such as Grb2,  **Crk** , and  **Crk**  L, which interact with guanine nucleotide exchange factors specific for the Ras family.  Ref: 9200440 J Immunol, 1997
- **Cbl**  is rapidly tyrosine phosphorylated in response to stimulation of a variety of cell surface receptors and becomes associated with a number of intracellular signalling molecules such as protein tyrosine kinases, phosphatidylinositol 3 kinase,  **Crk** , and 14 3 3 proteins through different protein interacting modules, leading to the formation of multimolecular signalling complexes.  Ref: 9720760 Cell Signal, 1998
**Neutral relationship**- In  **Cbl**  b T cells, the interaction between  **Crk**  L and C3G, and the activity of the small GTPase Rap1 are increased.  Ref: 12697763 J Biol Chem< MedlineTA>J Biol Chem, 1998
- Tyrosine phosphorylated  **Cbl**  binds to  **Crk**  after T cell activation.  Ref: 8683103 J Immunol, 1996
- It was previously reported that  **Cbl**  b associates with  **Crk**  L in Jurkat T cells.  Ref: 12697763 J Biol Chem< MedlineTA>J Biol Chem, 1996
- Furthermore, once phosphorylated,  **Cbl**  associated with  **Crk**  adaptor proteins.  Ref: 9129019 Blood, 1997
- We propose that tyrosine phosphorylation of membrane associated Cas and  **Cbl**  creates binding sites for the  **Crk**  SH2 domain and recruits  **Crk**  complexes to cellular membranes.  Ref: 8943292 J Biol Chem, 1996
- In Met transformed cells,  **Crk**  predominantly associates with the  **Cbl**  and Gab1docking proteins in a tyrosine phosphorylation dependent manner.  Ref: 11146548 Oncogene, 2000
- We identified two of these phosphoproteins as Cas and  **Cbl**  and showed that both bound to the  **Crk**  SH2 domain after BCR engagement.  Ref: 8943292 J Biol Chem, 1996
- After insulin dependent tyrosine phosphorylation, c  **Cbl**  specifically associates with endogenous c  **Crk**  and Fyn.  Ref: 9447983 Mol Cell Biol, 1998
- Here we show that phosphorylated  **Cbl**  recruits the CrkII  [ **CRK** ]  C3G complex to lipid rafts, where C3G specifically activates the small GTP binding protein TC10.  Ref: 11309621 Nature, 2001
- **Crk**  immunoprecipitates from activated T cell lysates contain tyrosine phosphorylated  **Cbl** .  Ref: 8683103 J Immunol, 1996
- Thus,  **Cbl**  b plays a negative role in  **Crk**  L C3G mediated Rap1 and LFA 1 activation in T cells.  Ref: 12697763 J Biol Chem< MedlineTA>J Biol Chem, 1996
- The Grb2 and the  **Crk**  L binding regions were mapped to the C terminus of  **Cbl**  b.  Ref: 10022120 Oncogene, 1999
- We also show that the  **Cbl**  EGF receptor complex is predominantly associated with CrkII  [ **CRK** ]  and is distinct to the Grb2 Shc Sos1 complex that associates with the EGF receptor.  Ref: 9416834 Oncogene, 1997
- Of the three  **Crk**  isoforms expressed in hematopoietic cells CrkL, CrkII  [ **CRK** ] , and CrkI, tyrosine phosphorylated  **Cbl**  binds preferentially to CrkL and CrkII  [ **CRK** ] .  Ref: 9129019 Blood, 1997
- Recent studies have also identified a novel signaling pathway initiated by the interaction of mammalian  **Cbl**  proteins with the SH2 domains of  **Crk**  adaptor molecules, which results in  **Cbl**  s linkage with C3G, a guanine nucleotide exchange protein for Rap1 family of small G proteins.  Ref: 9570294 Crit Rev Oncog, 1997
- We found that  **Cbl**  b associates with, and induces ubiquitin conjugation to  **Crk**  L, which requires a functional RING finger.  Ref: 12697763 J Biol Chem< MedlineTA>J Biol Chem, 1997
- BCR engagement causes  **Crk**  to bind to the Cas and  **Cbl**  docking proteins.  Ref: 9786933 J Biol Chem, 1998
- Although the exact mechanism of interaction between these molecules has not been fully defined, it has been generally accepted that  **Crk** , ZAP 70, and p85 interact with tyrosine phosphorylated  **Cbl** , which serves as a major scaffold protein in activated T lymphocytes.  Ref: 11418612 J Biol Chem, 2001
- The protein product of c  **cbl**  proto oncogene is known to interact with several proteins, including Grb2,  **Crk**  and PI3 kinase, and is thought to regulate signalling by many cell surface receptors.  Ref: 9367879 Biochem Biophys Res Commun, 1997
- The proto oncogene c  **Cbl**   [ **CBL** ]  is a positive regulator of Met induced MAP kinase activation a role for the adaptor protein  **Crk** .  Ref: 10962563 Oncogene, 2000
- T cell activation induces direct binding of the  **Crk**  adapter protein to the regulatory subunit of phosphatidylinositol 3 kinase p85 via a complex mechanism involving the  **Cbl**  protein.  Ref: 11418612 J Biol Chem, 2001
- After insulin dependent tyrosine phosphorylation, c  **Cbl**  specifically associates with fusion proteins containing the Src homology 2 SH2 domains of  **Crk**  and the Fyn tyrosine kinase, but not with fusion proteins containing the SH2 domains of either the p85 subunit of phosphatidylinositol 3 kinase or the tyrosine phosphatase SHPTP2 Syp.  Ref: 9210408 Biochem J, 1997
- Thus c  **Cbl**  behaves as a core protein, allowing the formation of a quaternary complex including,  **Crk**  II  [ **CRK** ] , PI 3 kinase and Grb2.  Ref: 9178909 Oncogene, 1997
- Furthermore, phosphopeptide binding studies revealed that the GST  **Crk**  SH2 domain binds to a tyrosine phosphorylated peptide corresponding to amino acids 770 781 of  **Cbl**  with high affinity.  Ref: 8683103 J Immunol, 1996
- Here we demonstrate that the  **Crk**  associated p116 phosphoprotein is not the  **Crk**  associated substrate Cas but, rather, is a protein product of the c  **cbl**  proto oncogene.  Ref: 8683103 J Immunol, 1996
- This association is mediated by the SH2 domain of  **Crk** , as evidenced by the interaction between  **Cbl**  and the fusion protein product of a glutathione S transferase GST expression construct encoding the  **Crk**  SH2 domain in vitro.  Ref: 8683103 J Immunol, 1996
- **Cbl**  serves as a substrate of both receptor and non receptor tyrosine kinases, and binds to adaptor proteins Grb2,  **Crk**  and the p85 subunit of PI 3 kinase.  Ref: 9675877 Int J Biochem Cell Biol, 1998
- **Crk**  is an SH2 and SH3 domain containing adaptor protein that has been reported to associate with p130cas, paxillin, c  **cbl** , c abl, Sos, and C3G in vitro.  Ref: 9348226 Endocrinology, 1997
- The  **Crk**  adapter proteins are assumed to play a role in T lymphocyte activation because of their induced association with tyrosine phosphorylated proteins, such as ZAP 70 and  **Cbl** , and with the phosphatidylinositol 3kinase regulatory subunit, p85, following engagement of the T cell antigen receptor.  Ref: 11418612 J Biol Chem, 2001
- We report here that c  **Cbl** , which is a Gab1 like docking protein, also becomes tyrosine phosphorylated in response to Met activation and serves as a docking molecule for various SH2 containing molecules, including  **Crk** .  Ref: 10962563 Oncogene, 2000
- The overexpression of a CAP mutant in which the SoHo domain had been deleted CAPDeltaSoHo prevented the translocation of  **Cbl**  to lipid rafts and subsequently blocked the recruitment of CrkII  [ **CRK** ]  and C3G.  Ref: 11481476 Proc Natl Acad Sci U S A, 2001

**Non-interactive relationship** (e.g. studied together, co-existance, homology, etc.)

- Erythropoietin and interleukin 3 activate tyrosine phosphorylation of  **CBL**  and association with  **CRK**  adaptor proteins.  Ref: 9129019 Blood, 1997
- We demonstrate here that CSF 1 exposure of stable transfectant CHO cells expressing the CSF 1 receptor induced the sustained tyrosine phosphorylation of c  **Cbl**  and its subsequent association with  **Crk**  II  [ **CRK** ]  and the p85 kDa subunit of the PI 3 kinase, while it constitutively associates with Grb2.  Ref: 9178909 Oncogene, 1997
- FL inducible binding of  **CBL**  to the CRKII  [ **CRK** ]  adaptor molecule is also demonstrated.  Ref: 9529123 Leukemia, 1998
- Other signalling molecules are also clearly involved, including p62 DOK, p95 VAV,  **CRK**  L, p12O  **CBL**  and focal adhesion proteins.  Ref: 9844814 Hematol Cell Ther, 1998
- Overexpression of the APS Y 618 F mutant in 3T3 L1 adipocytes blocked the insulin stimulated tyrosine phosphorylation of endogenous  **Cbl**  and binding to  **Crk** .  Ref: 11997497 Mol Cell Biol, 2002
- Because the signal from the Let 23 pathway affects the activation status of the Let 60 Ras homologue in C. elegans pathway, the activation dependent association between  **Crk**  and  **Cbl**  may represent another TCR generated signal leading to Ras related pathways.  Ref: 8683103 J Immunol, 1996
- Activation of nuclear factor of activated T cells NFAT and activating protein 1 AP 1 by oncogenic 70Z  **Cbl**  requires an intact phosphotyrosine binding domain but not  **Crk**  L or p85 phosphatidylinositol 3 kinase association.  Ref: 9988765 J Biol Chem, 1999
- This process is independent of PI 3 K, but requires the translocation of  **Cbl** ,  **Crk**  and C3G to the lipid raft.  Ref: 11309621 Nature, 2001
- It was found in complexes with many signaling proteins, including phosphoinositol PtdIns 3 kinase EC 2.7.1.137,  **Cbl** , GRB2, p130Cas and  **Crk** .  Ref: 12135478 Eur J Biochem, 2002
- Our data suggest that the inducible  **Cbl**   **Crk**  association is a proximal component of a signaling pathway downstream of multiple cytokine receptors.  Ref: 9129019 Blood, 1997
- CSF 1 stimulation induces the formation of a multiprotein complex including CSF 1 receptor, c  **Cbl** , PI 3 kinase,  **Crk**  II  [ **CRK** ]  and Grb2.  Ref: 9178909 Oncogene, 1997
- The presence of c  **cbl**   **Crk**  II  [ **CRK** ]  association may partly determine the signal specificities initiated by insulin and EGF.  Ref: 9348226 Endocrinology, 1997
- Several of these proteins were identified and include protein tyrosine kinase 2 also known as CAKbeta, RAFTK, and CADTK, pp125 focal adhesion tyrosine kinase, pp130  **Crk**  associated substrate, paxillin, and  **Cbl** .  Ref: 9582374 J Biol Chem, 1998
- This study therefore identifies a cytoskeletal role for c  **Cbl**  which may involve the regulation of  **Crk**  and Rac, and which is dependent on targeting of c  **Cbl**  to actin lamellae and the ability to recruit signalling protein s associated with its divergent SH2 domain.  Ref: 10633073 J Cell Sci, 2000
- We further show that  **Cbl**  is similarly capable of enhancing Met induced JNK activation, and several lines of experimentation suggests that it does so by interacting with  **Crk** .  Ref: 10962563 Oncogene, 2000
- When overexpressed in these cells, wild type APS but not an APS Y 618 F mutant facilitated the tyrosine phosphorylation of coexpressed  **Cbl**  and its association with  **Crk**  upon insulin stimulation.  Ref: 11997497 Mol Cell Biol, 2002
- Signaling factors that associate with activated erythropoietin Epo receptor complexes include phospholipase C gamma, phosphatidylinositol 3 kinase, SHIP, Shc, Grb2,  **Cbl** ,  **Crk**  l, HCP, Syp, and STAT5.  Ref: 9111017 J Biol Chem, 1997
- The amount of  **Cbl**  associated with CrkL and CrkII  [ **CRK** ]  exceeded the fraction of  **Cbl**  associated with Grb2 indicating that unlike other receptor systems, the  **Cbl**   **Crk**  association represents the dominant complex of  **Cbl**  in growth factor stimulated hematopoietic cells.  Ref: 9129019 Blood, 1997
- We conclude that 70Z  **Cbl**  induced NFAT AP1 activation requires the phosphotyrosine binding domain but not  **Crk**  L p85 PI3K association.  Ref: 9988765 J Biol Chem, 1999
- In addition, SDF 1alpha stimulation enhanced the association of  **cbl**  with PI 3 kinase,  **Crk**  L, and 14 3 3beta proteins.  Ref: 11157475 Blood, 2001
- We demonstrate here that PKA s activation of Rap1 was mediated by the Rap1 guanine nucleotide exchange factor C3G, the adaptor  **Crk**  L, the scaffold protein  **Cbl** , and the tyrosine kinase Src.  Ref: 11804588 Mol Cell, 2002
- EphA activation also induced  **Cbl**  association with  **Crk**  L and  **Crk**  II  [ **CRK** ]  adapters, but not the related Grb2 protein.  Ref: 12794130 J Immunol, 2003
- Formation of Shc Grb2 and  **Crk**  adaptor complexes containing tyrosine phosphorylated  **Cbl**  upon stimulation of the B cell antigen receptor.  Ref: 8710377 Oncogene, 1996
- We specifically examined a series of proteins known to be p210BCR ABL substrates, including rasGAP, Shc, SH PTP2, SH PTP1,  **CRK**  L,  **CBL** , paxillin, and STATs, and found that each were also tyrosine phosphorylated in response to p190BCR ABL and TEL ABL.  Ref: 8808688 Oncogene, 1996
- In the current study, we have examined cytokine dependent tyrosine phosphorylation of  **Cbl**  and its association with  **Crk**  proteins.  Ref: 9129019 Blood, 1997
- Here we show that, like TCR CD3 stimulation, stimulation of CD2 enhanced the association of c  **Cbl**  with both  **Crk**  L and the p85 subunit of phosphatidylinositol 3 kinase.  Ref: 11133830 Int Immunol, 2001
- Here we examined a regulatory role of  **Cbl**  b in  **Crk**  L C3G signaling pathway.  Ref: 12697763 J Biol Chem< MedlineTA>J Biol Chem, 2001
- Furthermore insulin stimulates the association of c  **Cbl**  with endogenous c  **Crk**  and Fyn in intact 3T3 L1 adipocytes.  Ref: 9210408 Biochem J, 1997
- Interestingly, EGF, but not insulin, stimulated tyrosine phosphorylation of c  **cbl**  and its association with  **Crk**  II  [ **CRK** ] .  Ref: 9348226 Endocrinology, 1997
- We demonstrate that 70Z  **Cbl**  shows increased basal and activation induced tyrosine phosphorylation and association with  **Crk**  L and p85 PI3K in Jurkat T cells.  Ref: 9988765 J Biol Chem, 1999
- However, unlike in other cell lines, CrkII  [ **CRK** ]  was not inducibly bound to  **Cbl**  in U87MG glioma cells.  Ref: 10595738 Jpn J Cancer Res, 1999
- We find that Rap1 and proposed regulators of Rap1 the proto oncogene product  **Cbl** ,  **Crk**  family adaptor proteins, and the Rap1 guanine nucleotide exchange factor C3G are expressed at equivalent levels in both double negative and double positive murine thymocytes.  Ref: 11069064 Eur J Immunol, 2000
- The coupling of Gab1, but not  **Cbl** , with  **Crk**  is retained in cells grown in suspension and enhances JNK activation.  Ref: 11146548 Oncogene, 2000
- There is an activation induced association of tyrosine phosphorylated  **Cbl**  with Grb2 and CrkL, respectively, but not CrkII  [ **CRK** ] .  Ref: 11263968 Biochem Biophys Res Commun, 2001
- Negative regulation of TCR mediated  **Crk**  L C3G signaling and cell adhesion by  **Cbl**  b.  Ref: 12697763 J Biol Chem< MedlineTA>J Biol Chem, 2001
- **Cbl**  not only interacts with Grb2, but also with the adaptor protein  **Crk** .  Ref: 8710377 Oncogene, 1996
- 70Z  **Cbl**  retains the ability to enhance NFAT and activating protein 1 AP1 activity in the absence of  **Crk**  L p85 PI3K association.  Ref: 9988765 J Biol Chem, 1999
- By immunofluorescence microscopy we have determined that c  **Cbl**  co localizes with the adaptor protein  **Crk**  to submembranous actin lamellae in NIH 3T3 fibroblasts.  Ref: 10633073 J Cell Sci, 2000
- **Cbl**  b deficiency does not affect  **Crk**  L stability, but its association with C3G.  Ref: 12697763 J Biol Chem< MedlineTA>J Biol Chem, 2000
